# Retinal nerve fiber layer thinning as a novel fingerprint for cardiovascular events: results from the prospective cohorts in UK and China

**DOI:** 10.1186/s12916-023-02728-7

**Published:** 2023-01-18

**Authors:** Yanping Chen, Yixiong Yuan, Shiran Zhang, Shaopeng Yang, Junyao Zhang, Xiao Guo, Wenyong Huang, Zhuoting Zhu, Mingguang He, Wei Wang

**Affiliations:** 1 State Key Laboratory of Ophthalmology, Zhongshan Ophthalmic Center, Sun Yat-sen University, Guangdong Provincial Key Laboratory of Ophthalmology and Visual Science, Guangdong Provincial Clinical Research Center for Ocular Diseases, Guangzhou, China; 2 Centre for Eye Research Australia, Royal Victorian Eye and Ear Hospital, University of Melbourne. Level 7, 32 Gisborne Street, East Melbourne, VIC 3002 Australia

**Keywords:** Cardiovascular disease, Retinal fingerprint, Retinal nerve fiber layer, Optical coherence tomography, Multi-ethnic cohort

## Abstract

**Background:**

Retinal structural abnormalities have been found to serve as biomarkers for cardiovascular disease (CVD). However, the association between retinal nerve fiber layer (RNFL) thickness and the incidence of CVD events remains inconclusive, and relevant longitudinal studies are lacking. Therefore, we aimed to examine this link in two prospective cohort studies.

**Methods:**

A total of 25,563 participants from UK Biobank who were initially free of CVD were included in the current study. Another 635 participants without retinopathy at baseline from the Chinese Guangzhou Diabetes Eye Study (GDES) were adopted as the validation set. Measurements of RNFL thickness in the macular (UK Biobank) and peripapillary (GDES) regions were obtained from optical coherence tomography (OCT). Adjusted hazard ratios (HRs), odd ratios (ORs), and 95% confidence intervals (CI) were calculated to quantify CVD risk.

**Results:**

Over a median follow-up period of 7.67 years, 1281 (5.01%) participants in UK Biobank developed CVD events. Each 5-μm decrease in macular RNFL thickness was associated with an 8% increase in incident CVD risk (HR = 1.08, 95% CI: 1.01–1.17, *p* = 0.033). Compared with participants in the highest tertile of RNFL thickness, the risk of incident CVD was significantly increased in participants in the lowest thickness tertile (HR = 1.18, 95% CI: 1.01–1.38, *p* = 0.036). In GDES, 29 (4.57%) patients developed CVD events within 3 years. Lower average peripapillary RNFL thickness was also associated with a higher CVD risk (OR = 1.35, 95% CI: 1.11–1.65, *p* = 0.003). The additive net reclassification improvement (NRI) was 21.8%, and the absolute NRI was 2.0% by addition of RNFL thickness over the Framingham risk score. Of 29 patients with incident CVD, 7 were correctly reclassified to a higher risk category while 1 was reclassified to a lower category, and 21 high risk patients were not reclassified.

**Conclusions:**

RNFL thinning was independently associated with increased incident cardiovascular risk and improved reclassification capability, indicating RNFL thickness derived from the non-invasive OCT as a potential retinal fingerprint for CVD event across ethnicities and health conditions.

**Trial registration:**

ISRCTN 15853192

**Supplementary Information:**

The online version contains supplementary material available at 10.1186/s12916-023-02728-7.

## Background

Cardiovascular disease (CVD) is one of the greatest medical challenges worldwide. As the leading cause of morbidity and mortality, CVD affects more than 500 million people and accounts for one third of all deaths globally, posing a substantial socioeconomic burden on the public health system [1–3]. Most CVDs can be prevented by addressing behavioral risk factors. Unfortunately, many individuals with CVD remain undiagnosed until life-threatening events occur. Thus, identifying individuals at the highest risk of developing cardiovascular event at an early stage is pivotal for the tailoring of timely interventions to preventing CVD and its related complications.

Substantial advances have been achieved concerning the pathogenesis of CVD due to various omics techniques that have identified many biomarkers [4–6]. In contrast, the markers currently used for CVD risk stratification are non-specific (e.g., age and smoking), measures target/end organ damage (e.g., serum creatinine levels and albuminuria), or have low precision (e.g., blood pressure and LDL cholesterol levels). Furthermore, the ability of these methods to track changes in CVD risk over time and in response to treatment remains unclear. Thus, the currently established indicators of CVD risk cannot be used to accurately quantify individual risk. There is an urging need to explore novel indicators which could provide increasingly accurate and personalized risk assessments.

The retina serves as a unique window of cardiovascular system because of their sharing similar embryonic origin, anatomical structure, and blood supply [7, 8]. Retinal optical coherence tomography (OCT) enables fast, ultra-high resolution, and automatic segmentation of individual retinal layers. Using OCT, alterations of retinal nerve fiber layer (RNFL) thickness have been implicated in cardiovascular health, generating new insights into the role of retinal fingerprints in the prediction of CVD. However, emerging evidence has yielded conflicting results. Some studies reported that localized RNFL thinning was strongly correlated with the presence of ischemic stroke in a hospital-based populations [9], and reduced RNFL thickness was observed in coronary heart disease and heart failure [10–12]. Shin et al. noted that Korean patients with thinner RNFL had a higher predicted 10-year cardiovascular risk assessed by atherosclerotic cardiovascular disease (ASCVD) risk score, compared with those without RNFL defects [13]. However, other studies reported no such correlations [14, 15]. This discrepancy might be associated with the cross-sectional nature of these studies, the fact that most merely focused on localized changes in the RNFL, the small sample size of the hospital patients, and the fact that confounding factors for CVD were not adjusted for. To date, prospective population-based cohort study focusing on the longitudinal relationship of RNFL thickness in both macular and peripapillary regions with the total cardiovascular burden is scarce.

Therefore, the objective of this study was to prospectively explore the association between macular RNFL thickness and CVD risk using data from the UK Biobank cohort in the European general population. Furthermore, given that only macular RNFL thickness was available in UK Biobank, we furtherly aimed to validate the association of peripapillary RNFL thickness with CVD onset and its reclassification value using data from the Guangzhou Diabetes Eye Study (GDES) cohort in the Chinese diabetic population.

## Methods

### Study design and population

The UK Biobank is a large population-based prospective cohort study that recruited over 500,000 participants aged 40 to 69 years between 2006 and 2010 throughout England, Scotland, and Wales. At baseline assessment, participants completed a touch-screen questionnaire covering demographic, socioeconomic, lifestyle, medication, systemic, and ocular disease information. Between 2009 and 2010, ocular assessments were introduced at six assessment centers, including visual acuity, autorefraction, intraocular pressure (IOP), and OCT examinations. Details on the overall study protocol and the protocols for each test have been described elsewhere [16].

**Fig. 1:**
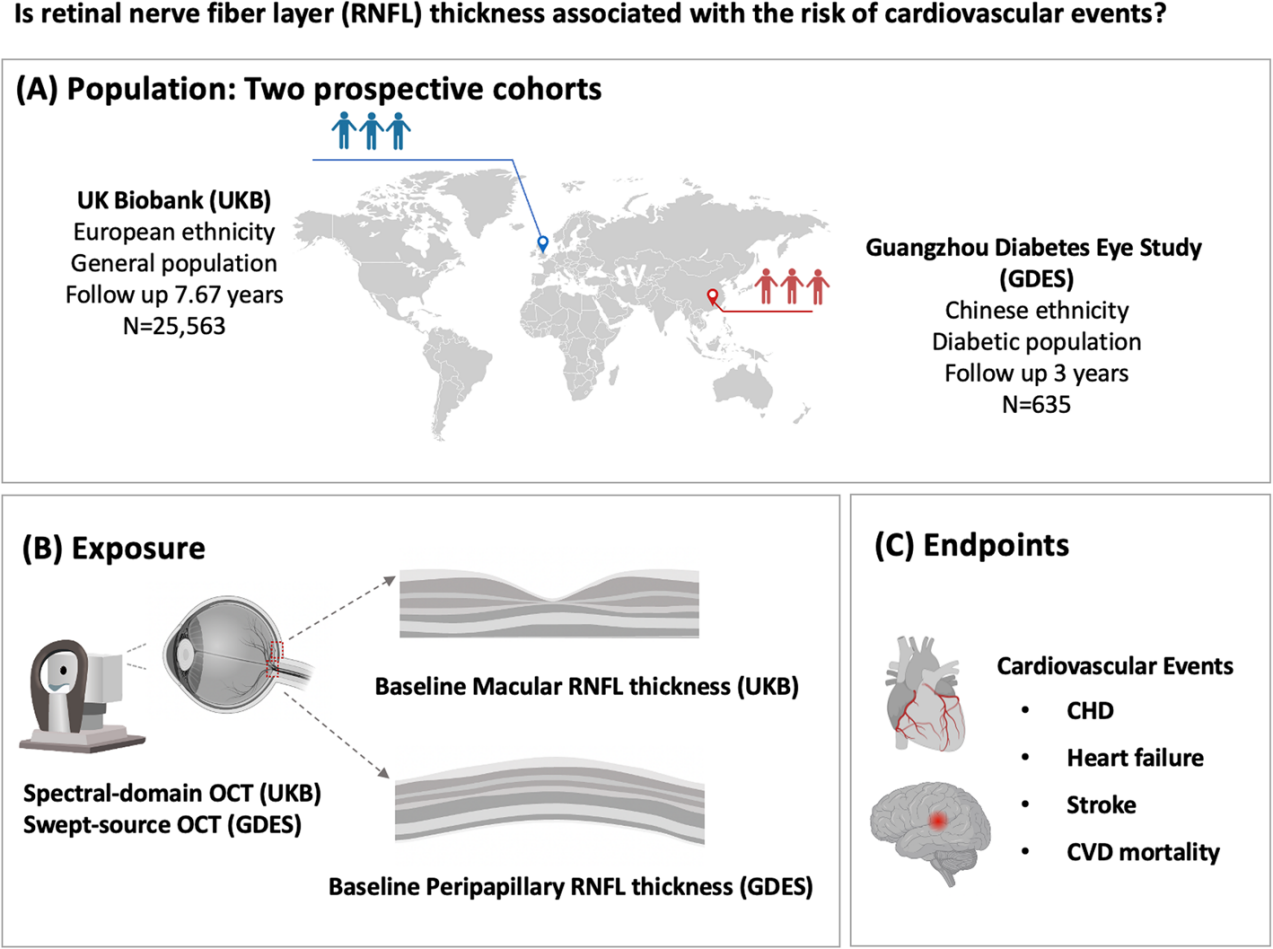
Workflow of this study. OCT, optical coherence tomography; CHD, coronary heart disease; CVD, cardiovascular disease

Figure 1 shows the workflow of the study. In the current study, a total of 67,287 participants from UK Biobank had available spectral domain OCT (SD-OCT) scans, among which 4959 with a history of CVD and/or cancer at the baseline were excluded. We also excluded those with poor-quality SD-OCT images (i.e., image quality score lower than 45, poor SD-OCT signal strength, poor centration certainty, or poor segmentation indicated by segmentation indicators as previously depicted in the UK Biobank) [17–19] and missing RNFL values (*N* = 15,634). To eliminate the impact of ocular conditions on RNFL assessment [19–21], the following ophthalmic states were further excluded (*N* = 15,691): high refractive error (± 6 diopters or greater [D]), visual acuity worse than 0.1 logarithm of the minimum angle of resolution (20/30 Snellen equivalent), IOP < 5 mmHg or > 22 mmHg, with self-reported glaucoma or retinal diseases. Finally, we excluded those with missing data on the main covariates of the present study at baseline (*N* = 5440). The remaining 25,563 participants were included in the current analysis. (Additional file 1: Figure S1)

### Assessment of RNFL thickness

In UK Biobank, SD-OCT was performed to obtain macular RNFL (mRNFL) thickness at baseline [18, 19, 22]. The SD-OCT imaging protocol was described in detail by Patel et al [23] according to the APOSTEL guidelines [24]. Briefly, OCT images were acquired using the Topcon 3D OCT-1000 Mk2 (Topcon, Tokyo, Japan) with a raster scan protocol, 6×6-mm^2^ in area, centered on the fovea (512 A-scans by 128 B-scans) in a dark room without pupil dilation. The Topcon Advanced Boundary Segmentation algorithm (Version 1.6.1.1) was then used to automatically segment and determine RNFL thickness, as previously described [19, 25] (Additional file 1: Figure S2). The mRNFL adopted in this study was the average thickness of RNFL across the ETDRS (Early Treatment Diabetic Retinopathy Study) 9 subregions with a diameter of 6 mm in a circle centered on the fovea in the macula [26].

### Definition of cardiovascular events

In UK Biobank, CVD was determined using data from hospital admissions and death registers. At the time of analysis, health-related outcome data was available up until December 31, 2017; thus, we used this date as the end of follow-up or the date of CVD incidence diagnosis or death from CVD, whichever occurred first. Incident CVD was defined using the following ICD 10 (International Classification of Diseases, 10th revision) codes: I20-25, I50, and I60-64.

### Ascertainment of covariates

CVD has been reported to be associated with various potential confounders according to previous studies, which have been included as covariates in the present analysis [27–30]. These factors included age, sex (male, female), ethnicity (white, non-white), household income (<£18,000, £18,000–30,999, £31,000–51,999, £52,000–100,000, > £100,000), education qualification (O levels, CSEs, GCSEs; A/AS levels; professional qualifications, NVQ, HND, HNC, college or university degree), Townsend deprivation index (least, second, third, most deprived quartile), body mass index (BMI calculated as body weight in kilograms divided by height squared; normal [18.5 kg/m^2^ < BMI < 25 kg/m^2^], overweight [25 kg/m^2^ < BMI < 30 kg/m^2^], obesity [> 30 kg/m^2^]), systolic blood pressure (SBP), diastolic blood pressure (DBP), HbA1c, fasting glucose, smoking status (never, ever/current), drinking status (never, ever/current), moderate-to-vigorous physical activity (least, second, third, most MVPA quartile) [31], hypertension (yes, no), diabetes (yes, no), antihypertension drug use (yes, no), insulin usage (yes, no), lipid-lowering drug use (yes, no). In addition, assessment center was also considered a covariate due to the imbalanced incident CVD events and health-related factors in different centers [32]. Given that several ocular measurements are associated with RNFL thickness [19, 33], spherical equivalent refraction (SER, calculated as sphere plus half of cylinder), IOP and image quality score were further considered as covariates. The definitions of variables are provided in Additional file 1: Table S1 with the UK Biobank Data field number and Data code.

### Association validated in the GDES cohort

In the present study, we furtherly validated the association of RNFL thickness with CVD onset in the Chinese ethnicity. The GDES is an ongoing community-based cohort study that includes type 2 diabetic patients recruited in 2017 in Guangzhou, China [34]. A total of 635 participants with the same eligibility criteria as participants from the UK Biobank and without any diabetic retinopathy at baseline were finally included. Swept Source OCT (SS-OCT, DRI OCT Triton; Topcon, Japan) was used to obtain the RNFL thickness at baseline. The scanning mode was 3.4 mm circle scan centered at the disc, and the built-in software (IMAGEnet 6, Version 1.22) was utilized for automatic retinal segmentation. To avoid the impact of hyperglycemia on macular RNFL measurement, the average peripapillary RNFL (pRNFL) thickness was assessed. The pRNFL thickness was also evaluated in four quadrants, i.e., inferior, superior, temporal, and nasal. Besides, in order to reduce the effect of diabetes on RNFL thickness, the related factors including age, HbA1c, duration of diabetes, and insulin usage were adjusted [35, 36]. CVD onset was defined as the development of coronary heart disease, heart failure, stroke, or related mortality in participants free of any CVD at baseline through the medical records and standard questionnaires for family members.

### Ethics, consent, and permissions

This study was reviewed and approved by the National Information Governance Board for Health and Social Care and the NHS North West Multicenter Research Ethics Committee (11/NW/0382), the Biobank consortium (application no. 62489), and ethics committee of Zhongshan Ophthalmic Center (reference no. 2017KYPJ094). All participants provided written informed consent for the study. The study was performed in accordance with the tenets of the Declaration of Helsinki and reported according to STROBE guideline.

### Statistical analysis

If both eyes of a patient were eligible for the inclusion criteria, one eye was chosen at random. Baseline characteristics were presented as number (percentage) for categorical variables and mean (standard deviation [SD]) for quantitative variables. The unpaired *t*-test or analysis of variance test was used to compare continuous variables, and the Pearson chi-squared test or Fisher’s exact test was used to compare categorical variables.

Participants were classified into three groups based on tertiles of baseline RNFL thickness, with those in the highest tertile (the thickest tertile) serving as the reference group. In the UK Biobank population, the log-rank test was used in the comparison of incidence rate of CVD among the mRNFL tertiles groups. Cox proportional hazard models were run to test the association between mRNFL thickness and incident CVD events. The association of both per 5-μm decrease in mRNFL thickness and its tertiles with incident CVD risk were assessed. Model 1 was a univariate model. Candidate variables with a *p* value < 0.10 in model 1 were selected for model 2. All variables met the proportional hazards assumption in the Cox models by Kaplan–Meier plot graphical assessment. Hazard ratios (HRs) and 95% confidence intervals (CIs) were computed to evaluate the degree of associations. In the GDES population, logistic regression models were used to assess the associations of pRNFL thickness with cumulative CVD onset during 3-year period. The odds ratios (ORs) and 95% CI were calculated to quantify the degree of associations between pRNFL parameters and CVD risk. In addition, the reclassification value of the average pRNFL thickness over the Framingham Risk Score (FRS) [4] was evaluated by additive and absolute net reclassification improvement (NRI) and integrated discrimination improvement (IDI) [37]. NRI and IDI were used to quantify whether pRNFL thickness as a novel biomarker tends to increase risk categories or predicted risk for patients with occurrence of CVD events and decrease risk categories or predicted risk for patients without incident CVDs [38, 39]. Categories of 3-year CVD risk were < 15%, 15 to 30%, and > 30% [40].

Statistical analysis was conducted using Stata (version 17, StataCorp, Texas, USA). A two-sided *p* value < 0.05 was defined as statistically significant.

## Results

### Baseline characteristics

In UK Biobank, among the included 25,563 participants, the mean age was 55.25 (8.20) years and 13,845 (54.2%) were female. Most of the participants (90.8%) were of white ethnicity. Participants with a thinner mRNFL thickness at baseline were more likely to be female (lowest vs. highest thickness tertile, 5048 [59.0%] vs. 4178 [49.2%], *p* < 0.001) and less likely to have hypertension (lowest vs. highest thickness tertile, 1875 [21.9%] vs. 2312 [27.2%], *p* < 0.001) or diabetes (lowest vs. highest thickness tertile, 271 [3.2%] vs. 406 [4.8%], *p* < 0.001) at baseline and less likely to use antihypertension drugs (lowest vs. highest thickness tertile, 1216 [14.2%] vs. 1637 [19.3%], *p* < 0.001) or lipid-lowering drugs (lowest vs. highest thickness tertile, 950 [11.1%] vs. 1336 [15.7%], *p* < 0.001) (Table 1).

**Table 1:** Baseline characteristics of the study population in UK Biobank stratified by mRNFL thickness tertiles

Characteristics	Total	Tertile of mRNFL thickness			***p***-value
		Highest	Middle	Lowest	
No.	25,563	8495	8515	8553	−
Age, years	55.25 (8.20)	56.32 (8.17)	55.11 (8.17)	54.33 (8.15)	**< 0.001**
Female	13,845 (54.2)	4178 (49.2)	4619 (54.3)	5048 (59.0)	**< 0.001**
Ethnicity					**< 0.001**
White	23,199 (90.8)	7534 (88.7)	7730 (90.8)	7935 (92.8)	
Non-white	2197 (8.6)	899 (10.6)	737 (8.7)	561 (6.6)	
Missing	167 (0.7)	62 (0.7)	48 (0.6)	57 (0.7)	
Assessment center					**< 0.001**
Sheffield	7040 (27.5)	2347 (27.6)	2312 (27.2)	2381 (27.8)	
Liverpool	1933 (7.6)	648 (7.6)	637 (7.5)	648 (7.6)	
Hounslow	4738 (18.5)	1698 (20.0)	1599 (18.8)	1441 (16.9)	
Croydon	6396 (25.0)	1893 (22.3)	2126 (25.0)	2377 (27.8)	
Birmingham	5411 (21.2)	1889 (22.2)	1828 (21.5)	1694 (19.8)	
Swansea	45 (0.2)	20 (0.2)	13 (0.2)	12 (0.1)	
Household income					**< 0.001**
< £18,000	3964 (15.5)	1468 (17.3)	1283 (15.1)	1213 (14.2)	
£18,000–30,999	5258 (20.6)	1886 (22.2)	1744 (20.5)	1628 (19.0)	
£31,000–51,999	6101 (23.9)	1963 (23.1)	2034 (23.9)	2140 (24.6)	
£52,000–100,000	5323 (20.8)	1572 (18.5)	1845 (21.7)	1906 (22.3)	
> £100,000	1613 (6.3)	473 (5.6)	521 (6.1)	619 (7.2)	
Missing	3304 (12.9)	1133 (13.3)	1088 (12.8)	1083 (12.7)	
Education level					**< 0.001**
O levels, CSEs, GCSEs	7408 (29.0)	2728 (32.1)	2460 (28.9)	2220 (26.0)	
A/AS levels	1570 (6.1)	485 (5.7)	527 (6.2)	558 (6.5)	
Professional qualifications	16,287 (63.7)	5164 (60.8)	5438 (63.9)	5685 (66.5)	
Missing	298 (1.2)	118 (1.4)	90 (1.1)	90 (1.1)	
Townsend index					**0.002**
Least deprived quartile	5372 (21.0)	1715 (20.2)	1801 (21.2)	1856 (21.7)	
Second quartile	6013 (23.5)	1913 (22.5)	2066 (24.3)	2034 (23.8)	
Third quartile	7160 (28.0)	2412 (28.4)	2399 (28.2)	2349 (27.5)	
Most deprived quartile	6992 (27.4)	2448 (28.8)	2242 (26.3)	2302 (26.9)	
Missing	26 (0.1)	7 (0.08)	7 (0.08)	12 (0.1)	
SBP, mmHg	132.0 (17.7)	133.6 (17.7)	131.6 (17.5)	130.7 (17.7)	**< 0.001**
DBP, mmHg	79.82 (9.99)	80.46 (9.95)	79.58 (9.95)	79.43 (10.04)	**< 0.001**
HbA1c, mmol/mol	35.52 (5.86)	35.95 (6.43)	35.41 (5.56)	35.20 (5.54)	**< 0.001**
Fasting glucose, mmol/L	5.10 (0.95)	5.15 (1.05)	5.09 (0.89)	5.07 (0.89)	**< 0.001**
Body mass index					**< 0.001**
Normal	8508 (33.3)	2613 (30.8)	2832 (33.3)	3063 (35.8)	
Overweight	10,934 (42.8)	3695 (43.5)	3652 (42.9)	3587 (41.9)	
Obesity	6002 (23.5)	2138 (25.2)	1994 (23.4)	1870 (21.9)	
Missing	119 (0.5)	49 (0.6)	37 (0.4)	33 (0.4)	
Smoking status					**0.001**
Never	8744 (34.2)	2984 (35.1)	2851 (33.5)	2909 (34.0)	
Ever/current	2492 (9.8)	896 (10.6)	813 (9.6)	783 (9.2)	
Missing	14,327 (56.1)	4615 (54.3)	4851 (57.0)	4861 (56.8)	
Drinking status					**0.002**
Never	840 (3.3)	289 (3.4)	284 (3.3)	267 (3.1)	
Ever/current	23,532 (92.1)	7749 (91.2)	7856 (92.3)	7927 (92.7)	
Missing	1191 (4.7)	457 (5.4)	375 (4.4)	359 (4.2)	
MVPA					**0.042**
Least MVPA quartile	5383 (21.1)	1823 (21.5)	1772 (20.8)	1788 (20.9)	
Second MVPA quartile	5446 (21.3)	1762 (20.7)	1857 (21.8)	1827 (21.4)	
Third MVPA quartile	5025 (19.7)	1614 (19.0)	1653 (19.4)	1758 (20.6)	
Most MVPA quartile	5261 (20.6)	1771 (20.9)	1798 (21.1)	1692 (19.8)	
Missing	4448 (17.4)	1525 (18.0)	1435 (16.9)	1488 (17.4)	
Hypertension at baseline					**< 0.001**
No	19,326 (75.6)	6183 (72.8)	6465 (75.9)	6678 (78.1)	
Yes	6237 (24.4)	2312 (27.2)	2050 (24.1)	1875 (21.9)	
Diabetes at baseline					**< 0.001**
No	24,591 (96.2)	8089 (95.2)	8220 (96.5)	8282 (96.8)	
Yes	972 (3.8)	406 (4.8)	295 (3.5)	271 (3.2)	
Antihypertension drug use					**< 0.001**
No	21,342 (83.5)	6858 (80.7)	7147 (83.9)	7337 (85.8)	
Yes	4221 (16.5)	1637 (19.3)	1368 (16.1)	1216 (14.2)	
Insulin usage					0.305
No	25,434 (99.5)	8449 (99.5)	8467 (99.4)	8518 (99.6)	
Yes	129 (0.5)	46 (0.5)	48 (0.6)	35 (0.4)	
Lipid-lowering drug use					**< 0.001**
No	22,181 (86.8)	7159 (84.3)	7419 (87.1)	7603 (88.9)	
Yes	3382 (13.2)	1336 (15.7)	1096 (12.9)	950 (11.1)	
SER, diopter	− 0.04 (1.90)	0.31 (1.88)	0.02 (1.80)	− 0.47 (1.93)	**< 0.001**
Intraocular pressure, mmHg	15.19 (2.94)	15.21 (2.97)	15.12 (2.95)	15.23 (2.89)	**0.027**
Image quality score	69.52 (8.68)	68.80 (9.06)	69.82 (8.60)	69.94 (8.32)	**< 0.001**

Data are presented as No. (percentage of participants) for categorical variables or mean (standard deviation [SD]) for continuous valuables. *mRNFL* macular retinal nerve fiber layer, *SBP* systolic blood pressure, *DBP* diastolic blood pressure, *MVPA* moderate-to-vigorous physical activity, *SER* spherical equivalent refraction

During a median follow-up duration of 7.67 (interquartile range 7.57–7.82) years, 1281 (5.01%) participants developed CVD events. Participants with incident CVD were older than those without CVD, with fewer female participants. There were significant differences in ethnicity, assessment center, household income, educational qualification, SBP, DBP, HbA1c, fasting glucose, BMI, smoking status, MVPA, history of hypertension or diabetes, antihypertensive drugs, insulin usage, lipid-lowering drugs usage, SER, and IOP between the two groups (Table 2). Participants with incident CVD had a thinner baseline RNFL compared with those without CVD (27.83 [4.16] μm vs. 28.55 [4.25] μm, *p* < 0.001).

**Table 2:** Baseline characteristics of the study population in UK Biobank stratified by incident CVD

Characteristics	Non-CVD group	CVD group	***p*** value
No.	24,282	1281	-
Age, years	55.02 (8.20)	59.59 (7.02)	**< 0.001**
Female	13,388 (55.1)	457 (35.7)	**< 0.001**
Ethnicity			**0.032**
White	22,013 (90.7)	1186 (92.6)	
Non-white	2112 (8.7)	85 (6.6)	
Missing	157 (0.7)	10 (0.8)	
Assessment center			**<0.001**
Sheffield	6655 (27.4)	385 (30.1)	
Liverpool	1827 (7.5)	106 (8.3)	
Hounslow	4561 (18.8)	177 (13.8)	
Croydon	6106 (25.2)	290 (22.6)	
Birmingham	5091 (21.0)	320 (25.0)	
Swansea	42 (0.2)	3 (0.2)	
Household income			**< 0.001**
< £18,000	3700 (15.2)	264 (20.6)	
£18,000–30,999	4971 (20.5)	287 (22.4)	
£31,000–51,999	5846 (24.1)	255 (19.9)	
£52,000–100,000	5111 (21.1)	212 (16.6)	
> £100,000	1555 (6.4)	58 (4.5)	
Missing	3099 (12.8)	205 (16.0)	
Education qualification			**< 0.001**
O levels, CSEs, GCSEs	6923 (28.5)	485 (37.9)	
A/AS levels	1523 (6.3)	47 (3.7)	
Professional qualifications	15,566 (64.1)	721 (56.3)	
Missing	270 (1.1)	28 (2.2)	
Townsend deprivation index			0.311
Least deprived quartile	5077 (20.9)	295 (23.0)	
Second quartile	5718 (23.6)	295 (23.0)	
Third quartile	6805 (28.0)	355 (27.7)	
Most deprived quartile	6656 (27.4)	336 (26.2)	
Missing	26 (0.1)	0	
Systolic blood pressure, mmHg	131.60 (17.59)	138.90 (18.05)	**< 0.001**
Diastolic blood pressure, mmHg	79.69 (9.96)	82.26 (10.38)	**< 0.001**
HbA1c, mmol/mol	35.41 (5.67)	37.53 (8.51)	**< 0.001**
Fasting glucose, mmol/L	5.09 (0.92)	5.26 (1.31)	**< 0.001**
Body mass index			**< 0.001**
Normal	8234 (33.9)	274 (21.4)	
Overweight	10,360 (42.7)	574 (44.8)	
Obesity	5577 (23.0)	425 (33.2)	
Missing	111 (0.5)	8 (0.6)	
Smoking status			**< 0.001**
Never	8273 (34.1)	471 (36.8)	
Ever/current	2301 (9.5)	191 (14.9)	
Missing	13,708 (56.5)	619 (48.3)	
Drinking status			0.622
Never	792 (3.3)	48 (3.8)	
Ever/current	22,360 (92.1)	1172 (91.5)	
Missing	1130 (4.7)	61 (4.8)	
MVPA			**< 0.001**
Least MVPA quartile	5078 (20.9)	305 (23.8)	
Second MVPA quartile	5219 (21.5)	227 (17.7)	
Third MVPA quartile	4807 (19.8)	218 (17.0)	
Most MVPA quartile	5005 (20.6)	256 (20.0)	
Missing	4173 (17.2)	275 (21.5)	
Hypertension at baseline			**< 0.001**
No	18,566 (76.5)	760 (59.3)	
Yes	5716 (23.5)	521 (40.7)	
Diabetes at baseline			**< 0.001**
No	23,416 (96.4)	1175 (91.7)	
Yes	866 (3.6)	106 (8.3)	
Antihypertension drug use			**< 0.001**
No	20,441 (84.2)	901 (70.3)	
Yes	3841 (15.8)	380 (29.7)	
Insulin usage			**< 0.001**
No	24,175 (99.6)	1259 (98.3)	
Yes	107 (0.4)	22 (1.7)	
Lipid-lowering drug use			**< 0.001**
No	21,228 (87.4)	953 (74.4)	
Yes	3054 (12.6)	328 (25.6)	
SER, diopter	− 0.06 (1.90)	0.20 (1.90)	**< 0.001**
Intraocular pressure, mmHg	15.18 (2.94)	15.37 (2.90)	**0.024**
Image quality score	69.56 (8.67)	68.63 (8.78)	**< 0.001**
Average mRNFL thickness (μm)	28.55 (4.25)	27.83 (4.16)	**< 0.001**

Data are presented as No. (percentage of participants) for categorical variables or mean (standard deviation [SD]) for continuous valuables. *mRNFL* macular retinal nerve fiber layer, *MVPA* moderate-to-vigorous physical activity, *SER* spherical equivalent refraction

### Association between mRNFL thickness and CVD events

**Fig. 2:**
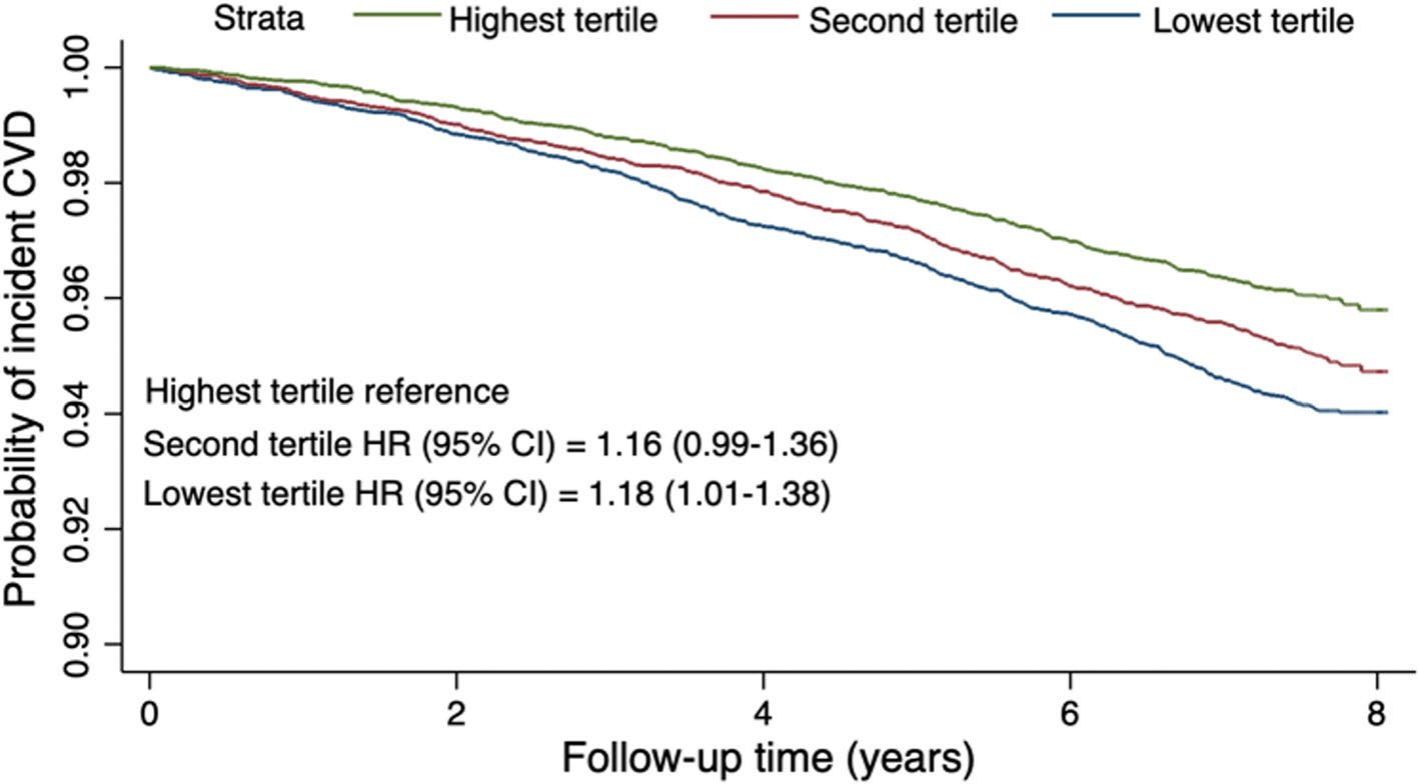
Adjusted Kaplan-Meier plot for CVD risk by mRNFL thickness tertiles in UK Biobank. Probability of incident CVD risk was shown over time for participants from UK Biobank with different mRNFL thickness tertiles. Lower tertile represents thinner mRNFL thickness. Plot was based on Cox proportion hazards regression models, adjusted for age, sex, ethnicity, assessment center, household income, Townsend deprivation index, SBP, HbA1c, fasting glucose, BMI, smoking status, drinking status, MVPA, history of hypertension and diabetes, antihypertensive drugs, insulin usage, lipid-lowering drugs, IOP, SER, and image quality score. Compared to participants with highest tertile of mRNFL thickness, CVD risk was comparable for those in the second tertile (HR = 1.16, 95% CI: 0.99–1.36, *p* = 0.065). The risk of incident CVD was significantly increased for participants in the lowest tertile of mRNFL thickness (HR = 1.18, 95% CI: 1.01–1.38, *p* = 0.036). CVD, cardiovascular disease; mRNFL, macular retinal nerve fiber layer; HR, hazard ratio; CI, confidence interval

The annual incidence rate of CVD was higher in patients with lower baseline mRNFL thickness (*p* log-rank test < 0.001; Additional file 1: Table S2). The Kaplan–Meier plot of participants with mRNFL thickness in each tertile was shown in Fig. 2. After adjusting for age, sex, ethnicity, assessment center, household income, Townsend deprivation index, SBP, HbA1c, fasting glucose, BMI, smoking status, drinking status, MVPA, history of hypertension and diabetes, antihypertensive drugs, insulin usage, lipid-lowering drugs, IOP, SER, and image quality score, each 5-μm decrease in baseline mRNFL thickness was associated with an 8% increase in incident CVD risk (HR = 1.08, 95% CI: 1.01–1.17, *p* = 0.033; Table 3). Compared with participants in the highest mRNFL thickness tertile, CVD risk was significantly increased by 18% for participants in the lowest thickness tertile (HR = 1.18, 95% CI: 1.01–1.38, *p* = 0.036). Additionally, mRNFL thickness tertiles showed a strong inverse association with incident CVD (*p*-value for trend = 0.043).

**Table 3:** Association between average mRNFL thickness and incident CVD in UK Biobank population

RNFL thickness	Model 1		Model 2	
	HR (95% CI)	***p*** value	HR (95% CI)	***p*** value
Per 5 μm decrease	1.22 (1.14–1.31)	**< 0.001**	1.08 (1.01–1.17)	**0.033**
Tertiles				
Highest	Reference	**−**	Reference	**−**
Second	1.26 (1.10–1.46)	**0.001**	1.16 (0.99–1.36)	0.065
Lowest	1.49 (1.30–1.70)	**< 0.001**	1.18 (1.01–1.38)	**0.036**
P for trend	**−**	**< 0.001**	**−**	**0.043**

*CVD* cardiovascular diseases, *mRNFL* macular retinal nerve fiber layer, *HR* hazard ratio, *CI* confidence interval

Model 1 was a univariate model

Model 2 was adjusted for age, sex, ethnicity, assessment center, household income, Townsend deprivation index, SBP, HbA1c, fasting glucose, BMI, smoking status, drinking status, MVPA, history of hypertension and diabetes, antihypertensive drugs, insulin usage, lipid-lowering drugs, IOP, SER, and image quality score

### Validation analysis in the GDES cohort

**Fig. 3:**
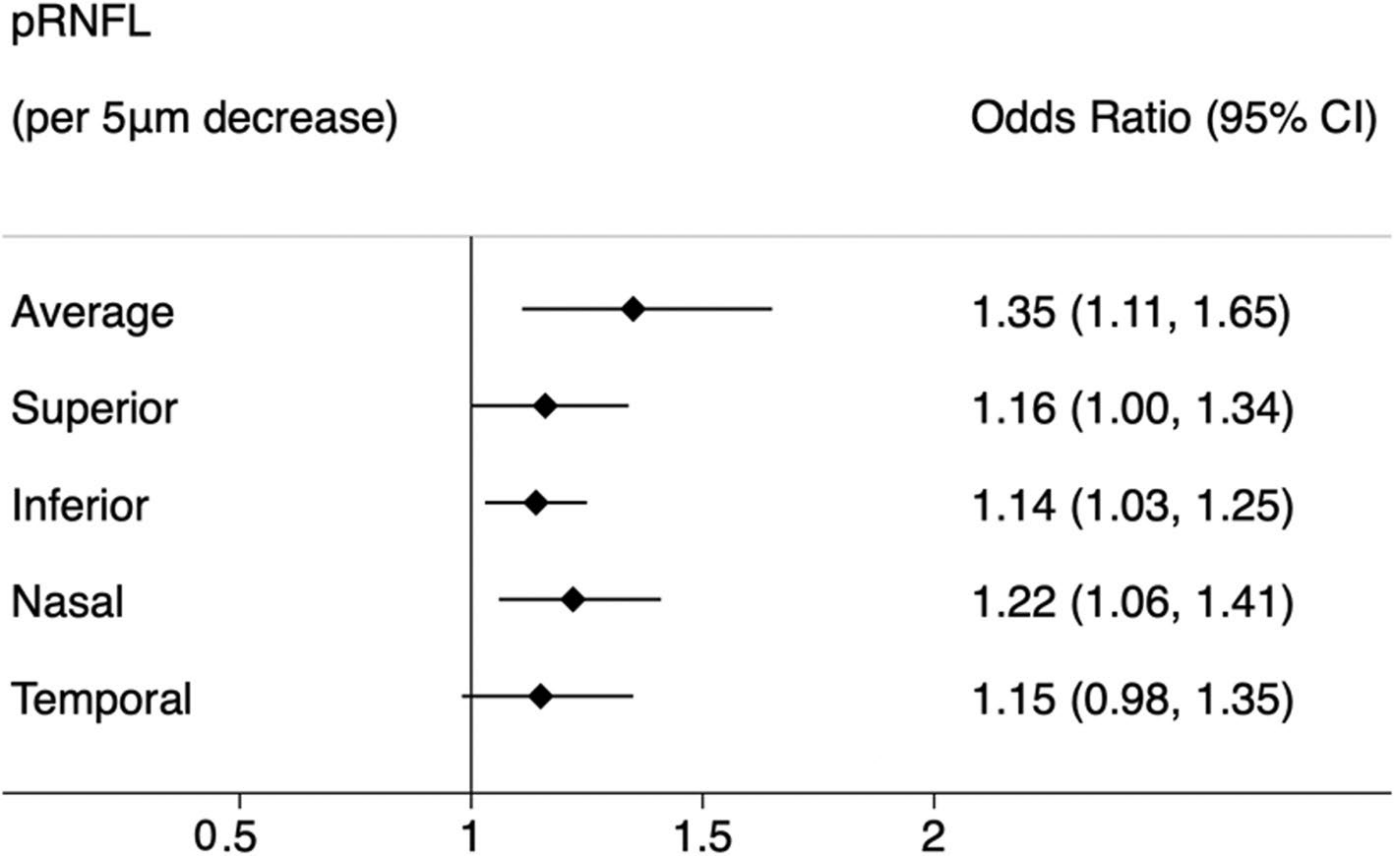
Association of pRNFL with incident CVD risk in GDES participants using logistic regression analysis. Multivariate model adjusted for age, sex, SBP, drinking status, duration of diabetes, HbA1c, insulin usage, axial length, and image quality score. pRNFL, peripapillary retinal nerve fiber layer; CVD, cardiovascular disease; SBP, systolic blood pressure; CI, confidence interval

In the GDES cohort, 29 (4.57%) of 635 patients developed CVD events during 3-year follow-up. The baseline characteristics and distribution of the pRNFL were shown in Additional file 1: Tables S3 and S4. The average pRNFL thickness was significantly lower in participants with incident CVD events than in those without CVD events (91.21 [20.14] μm vs. 110.41 [12.07] μm; *p* < 0.001). After adjusting for age, sex, SBP, drinking status, duration of diabetes, HbA1c, insulin usage, axial length, and image quality score, a lower baseline average pRNFL thickness was significantly associated with a higher CVD risk (OR = 1.35, 95% CI :1.11–1.65, *p* = 0.003; Fig. 3). Moreover, the inverse association between pRNFL thickness and incident CVD events were demonstrated in the four quadrants (Fig. 3).

### Reclassification ability of pRNFL thickness on CVDs

In the GDES cohort, the number of individuals reclassified using the model that included FRS and average pRNFL thickness were depicted in Table 4. Of 29 patients with incident CVDs, 7 were correctly reclassified to a higher risk category while 1 was reclassified to a lower category and 21 (72.4%) high risk patients were not reclassified. In people remained free of CVD events (*n* = 606), 16 were correctly reclassified to a lower risk category, and 9 were reclassified to a higher category. In all, 13 out of 635 (2.0%) participants were better classified by including pRNFL thickness. The addition of pRNFL thickness results in an additive NRI of 21.8% (*p* = 0.026) and an absolute NRI of 2.0%. Besides, these results were also confirmed by the assessment of IDI (13.1%, *p* = 0.011).

**Table 4:**
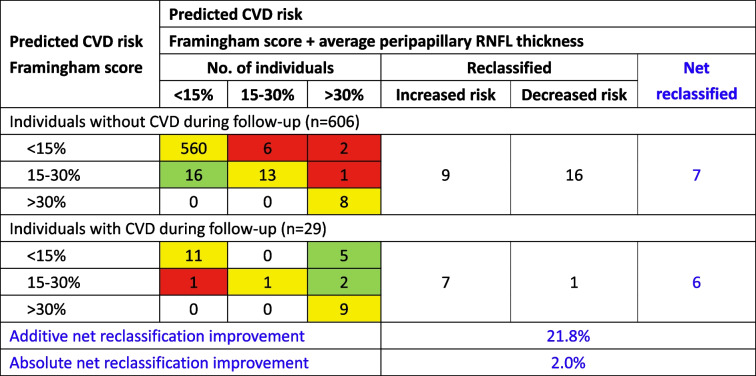
Risk reclassification ability of peripapillary RNFL thickness over the Framingham Risk Score for cardiovascular events in GDES cohort

The addition of peripapillary RNFL thickness to the Framingham Risk Score results in an additive net reclassification improvement (NRI) of 21.8% (*p* = 0.026) and an absolute NRI of 2.0%. The green cells represent patients correctly reclassified. The yellow cells represent risk stratification that is unchanged in the novel model. The red cells represent patients incorrectly reclassified. *RNFL* retinal nerve fiber layer, *CVD* cardiovascular disease

## Discussion

In a large population of more than 25,000 European individuals, we found that each 5-μm reduction in macular RNFL thickness was associated with an 8% increased risk of new-onset CVD, independent of other systemic and ocular factors. Patients in the lowest baseline RNFL thickness tertile had an 18% higher incident cardiovascular risk compared with those with highest RNFL thickness tertile. The association between reduced peripapillary RNFL thickness and increased CVD risk was further validated in the Chinese diabetic cohort. Furthermore, RNFL thickness improved the risk reclassification ability on the basis of Framingham risk valuables despite the small sample. Therefore, OCT-derived RNFL thinning is a potentially preclinical fingerprint of CVD events and could act as an early non-invasive surrogate for the future risk of major vascular diseases.

Our prospective findings are in line with previous cross-sectional studies (Additional file 1: Table S5) [9, 10, 12–15, 41–44]. Localized RNFL thinning assessed by fundus camera has been reported to be correlated with the presence of cerebrovascular diseases, including stroke and cerebral small vessel disease [9, 42]. Utilizing quantitative OCT assessment, further studies have reported that patients with ischemic heart diseases, such as coronary heart disease, serious heart failure, and congenital heart disease had a thinner RNFL compared to age- and sex- matched healthy participants [10, 12, 41]. Moreover, reduced RNFL thickness was also delineated at an earlier stage in the condition of established cardiovascular risk factors. Localized RNFL thinning was associated with the severity of asymptomatic carotid artery stenosis and arterial hypertension, in addition to retinal microvasculature abnormalities [43, 44]. In animal studies, chronic experimentally induced atherosclerosis and hypertension led to reduced visibility of RNFL with defect in rhesus monkeys [45]. Recently, a single-center study has demonstrated higher 10-year predicted cardiovascular risk by ASCVD score in patients with RNFL thinning [13]. However, this cross-sectional study only included individuals with high cardiovascular risk and merely focused on localized RNFL defect. Using a large-scale population-based cohort and a community-based cohort, we provided the first longitudinal evidence for the association of OCT-derived average RNFL thinning with the total burden of incident CVD events.

Although the exact pathophysiology of RNFL thinning in CVD remains unknown, it has been postulated to be due to the impairment of ocular circulation. The retina is supplied by the dual end arterioles, central retinal artery, and short posterior ciliary arteries, with autoregulatory mechanisms to maintain ocular perfusion. In the case of subclinical cardiovascular and cerebrovascular abnormalities, retinal vasoconstriction reduces blood supply to the retina in favor of adequate perfusion to the systemic circulation and important organs, leaving the inner retinal layers susceptible to ischemic damage [7]. It is therefore probable that microvascular pathologies, including atherosclerosis, arterial hypertension, increased rigidity and insufficient autoregulation, could be the main driver behind the reduction of RNFL thickness [46]. Moreover, retinal ischemia/reperfusion induced oxidative stress injury, cytokines release, and nerve fiber death might contribute to secondary RNFL thinning [47]. However, further studies are warranted to uncover the relevant underlying mechanisms.

The most notable advancement in ophthalmology was the advent of high-resolution OCT technology, a rapid, non-invasive and widely available imaging modality capable of producing a high-resolution cross-sectional image reflecting the near-histological tissue microstructure in vivo [48, 49]. As the only human tissue that allows direct non-invasive visualization of microvascular circulation and the central nervous system, the retina provides a unique window for documenting systemic diseases [48, 50, 51]. OCT examination is increasingly being routinely performed in hospital and community settings. The number of OCT scans increased 14-fold from 23,500 scans in 2008 to over 330,000 scans in 2016 at Moorfields Eye Hospital NHS Foundation Trust [52]. Furthermore, it has been reported that healthcare-seeking behavior for eye health has surpassed that for cardiovascular disease [49, 53], which provides OCT with an unprecedented opportunity to detect systemic disease, predict its onset, and quantify its severity and response to treatment. With the emergence of automated segmentation and precise quantification of individual retinal layers by deep learning algorithms [54], our discovery provides a novel insight into the role of OCT derived morphological RNFL abnormalities in the pathologies of cardiovascular events.

About 25% individuals in UK Biobank were excluded due to the poor image quality in the present analysis. This may be attributed to the poor patients’ cooperation and the inefficiency of the traditional SD-OCT system for dense volume scanning, the scanning speed of which was only 27 kHz per second without eye tracking function. However, novel SS-OCT system could reach a much faster scanning speed of up to 100 kHz per second, which brought faster, deeper, and clearer OCT images [55]. Moreover, the applied eye-tracking system could reduce eye motion artifacts and improve the signal-to-noise ratio [55]. In our GDES Chinese cohort, DRI Triton SS-OCT OCT was used, and the excluded rate due to the poor quality was less than 5%, which was comparable with another RNFL study [56]. More importantly, a recently devised algorithm could help adjust the effect of poor signal strength on OCT parameters evaluation [57]. Therefore, with the state-of-the-art modalities and standardized operations, the OCT images of poor quality will be reduced and more studies with more advanced OCT systems and algorithms are required to validate our findings in clinical practice.

Notably, both macular and peripapillary average RNFL thinning were independently associated with cardiovascular risk in European and Chinese populations. Moreover, the inclusion of RNFL thickness improved the reclassification abilities for CVD events over the classic FRS prediction model. Given the non-invasive nature of OCT and the high adherence rate of regular eye examination, RNFL thickness assessment from a single “eye-check” is an attractive alternative for assessing cardiovascular risk in the primary healthcare screening. In the community optometric practice, cases of RNFL thinning, unexplained by glaucoma, diabetic retinopathy, or other ocular conditions [19] should have concomitant cardiovascular risk factors considered and be considered for referral and further cardiovascular or neurological investigations. This measure is instructive in the early identification of patients with compromised cardiovascular health, translating to timely interventions targeting modifiable CVD risk factors, such as controlling hypertension, quitting tobacco and alcohol, regulating blood sugar and lipids, and moderately increasing physical activities [58, 59]. However, we should point out that the additive NRI of 21.8% was mainly based on the small sample with incident cardiovascular events. Twenty-one out of 29 patients with cardiovascular events could not be reclassified with the novel prediction model yet and the absolute NRI was 2.0%. Despite the improved prediction by the addition of pRNFL thickness, the small sample size may limit the power effect and more evidence from large-scale longitudinal studies is of necessity. Besides, further studies are needed to determine the cutoff value of reduced RNFL thickness in different age, sex groups for cardiovascular risk. This also includes more work to estimate the cost-effectiveness and acceptability of OCT application in community screenings.

To the best of our knowledge, this is the first prospective study to investigate the association between RNFL thickness and incident CVD events. The major strengths of this study were its large sample size, the relatively long follow-up period of nearly 8 years, the prospective design of two cohorts for comprehensive analysis of RNFL thickness within the macula and peripapillary regions and the rigorous findings after adjustments for numerous covariates. Furthermore, we have verified the generalization of our findings in European and Chinese ethnicities, in general and diabetic populations, and with different RNFL assessment devices (SD-OCT or SS-OCT). Nevertheless, we also acknowledged the limitations in this study. First, given that the incidence rates of each single cardiovascular event were low, the endpoint in our analysis was the total CVD burden combining incident coronary heart disease, heart failure, and stroke. Further studies are warranted to investigate the association between RNFL thickness and the specific CVD outcomes and further validate our findings. Second, although a wide range of demographic, lifestyle, and medication factors were adjusted, many covariates were self-reported, and thus, residual confounding possibly remains. Third, the association of RNFL thickness with the progression, treatment response, and prognosis of CVD could not be interpreted in the present study, which requires further clarification. Fourthly, despite the improved reclassification ability by the inclusion of pRNFL thickness over FRS, the sample size with incident CVD events was relatively small (only 29 patients) which may limit the statistical power. Future prospective studies with larger cardiovascular events sample are required to verify the prognostic value of RNFL thickness.

## Conclusions

In conclusion, RNFL thinning quantified by OCT were independent risk factors for CVD occurrence and improved reclassification capability, indicating RNFL thickness as a potential retinal fingerprint for cardiovascular events screening. Further prospective studies are needed to explore the predictive value of RNFL thickness for cardiovascular events progression, prognosis, and treatment response and to elucidate the possible biological mechanisms.

## Supplementary Information

**Additional file 1: Figure S1.** Flowchart showing inclusion and exclusion criteria of the population from UK Biobank. **Figure S2.** Macular OCT retinal layers segmentation in one sample OCT image from the UK Biobank. **Table S1.** Definition of variables in touchscreen questionnaire, verbal interview, and inpatient records of diagnosis. **Table S2.** Incidence rates of CVD Events in UK Biobank stratified by mRNFL thickness tertiles. **Table S3.** Baseline characteristics of participants in GDES stratified by incident CVD. **Table S4.** Distribution of baseline peripapillary RNFL (pRNFL) of participants in GDES stratified by incident CVD. **Table S5.** Summary of previous cross-sectional studies on RNFL and cardiovascular diseases.

## Data Availability

The dataset supporting the conclusion of the article is available in the UK Biobank (https://www.ukbiobank.ac.uk/). These data are available from the corresponding author on reasonable request and with permission of UK Biobank.

## Abbreviations

CVD: Cardiovascular disease
RNFL: Retinal nerve fiber layer
OCT: Optical coherence tomography
IOP: Intraocular pressure
SD: Standard deviation
HR: Hazard ratio
OR: Odd ratio
CI: Confidence interval
GDES: Guangzhou Diabetes Eye Study
ASCVD: Atherosclerotic cardiovascular disease
ETDRS: Early Treatment Diabetic Retinopathy Study
SBP: Systolic blood pressure
DBP: Diastolic blood pressure
BMI: Body mass index
MVPA: Moderate-to-vigorous physical activity
SER: Spherical equivalent refraction
mRNFL: Macular retinal nerve fiber layer
pRNFL: Peripapillary retinal nerve fiber layer
FRS: Framingham Risk Score
NRI: Net reclassification improvement
IDI: Integrated discrimination improvement
