# Supplementary material for: Retinal nerve fiber layer thinning as a novel fingerprint for cardiovascular events: results from the prospective cohorts in UK and China
Source: BMC Med. 2023 Jan 18;21:24. doi: 10.1186/s12916-023-02728-7 (PMC9850527; doi:10.1186/s12916-023-02728-7)
Supplement: Supplementary file 1 — Additional file 1: Figure S1. Flowchart showing inclusion and exclusion criteria of the population from UK Biobank. Figure S2. Macular OCT retinal layers segmentation in one sample OCT image from the UK Biobank. Table S1. Definition of variables in touchscreen questionnaire, verbal interview, and inpatient records of diagnosis. Table S2. Incidence rates of CVD Events in UK Biobank stratified by mRNFL thickness tertiles. Table S3. Baseline characteristics of participants in GDES stratified by incident CVD. Table S4. Distribution of baseline peripapillary RNFL (pRNFL) of participants in GDES stratified by incident CVD. Table S5. Summary of previous cross-sectional studies on RNFL and cardiovascular diseases. [file 12916_2023_2728_MOESM1_ESM.docx]

**Retinal nerve fiber layer thinning as a novel fingerprint for cardiovascular events: results from the prospective UK Biobank and a Chinese cohort**

**Running title:** RNFL Thinning Predicts Cardiovascular Events Risk

**Authors:** Yanping Chen, MD^1^*, Yixiong Yuan, MD^1^*, Shiran Zhang, MD^1^, Shaopeng Yang, MD^1^, Junyao Zhang, BBiomedSc^2^, Xiao Guo, MD^1^, Wenyong Huang, MD, PhD^1,^ Zhuoting Zhu, MD, PhD^2^†, Mingguang He, MD, PhD1,^2^†, Wei Wang, MD, PhD^1^†

**Affiliation and institute**

1. State Key Laboratory of Ophthalmology, Zhongshan Ophthalmic Center, Sun Yat-sen University, Guangdong Provincial Key Laboratory of Ophthalmology and Visual Science, Guangdong Provincial Clinical Research Center for Ocular Diseases, Guangzhou, China.

2. Centre for Eye Research Australia, Royal Victorian Eye and Ear Hospital, Melbourne, Australia.

*Co-first authors.

†Co-corresponding authors.

**Corresponding authors:**

Wei Wang, MD & PhD, Zhongshan Ophthalmic Center, State Key Laboratory of Ophthalmology, Sun Yat-sen University, Guangzhou, China. Email: wangwei@gzzoc.com

Mingguang He, MD & PhD & FRANZCO, NHMRC Leadership Fellow, Professor of Ophthalmic Epidemiology, University of Melbourne, Centre for Eye Research Australia. Level 7, 32 Gisborne Street, East Melbourne, VIC 3004, Australia.

Email: mingguang.he@unimelb.edu.au | M: 0458118880

Zhuoting Zhu, MD&PhD, Centre for Eye Research Australia, University of Melbourne. Level 7, 32 Gisborne Street, East Melbourne, VIC 3002, Australia, Email: lisa.zhu@unimelb.edu.au

**Word count: Abstract: 338; Whole paper: 3932.**

**Figures: 3**

**Tables: 4**

**Supplementary Figures: 2**

**Supplementary Tables: 5**

**Figure S1.** Flowchart showing inclusion and exclusion criteria of the population from UK Biobank

**
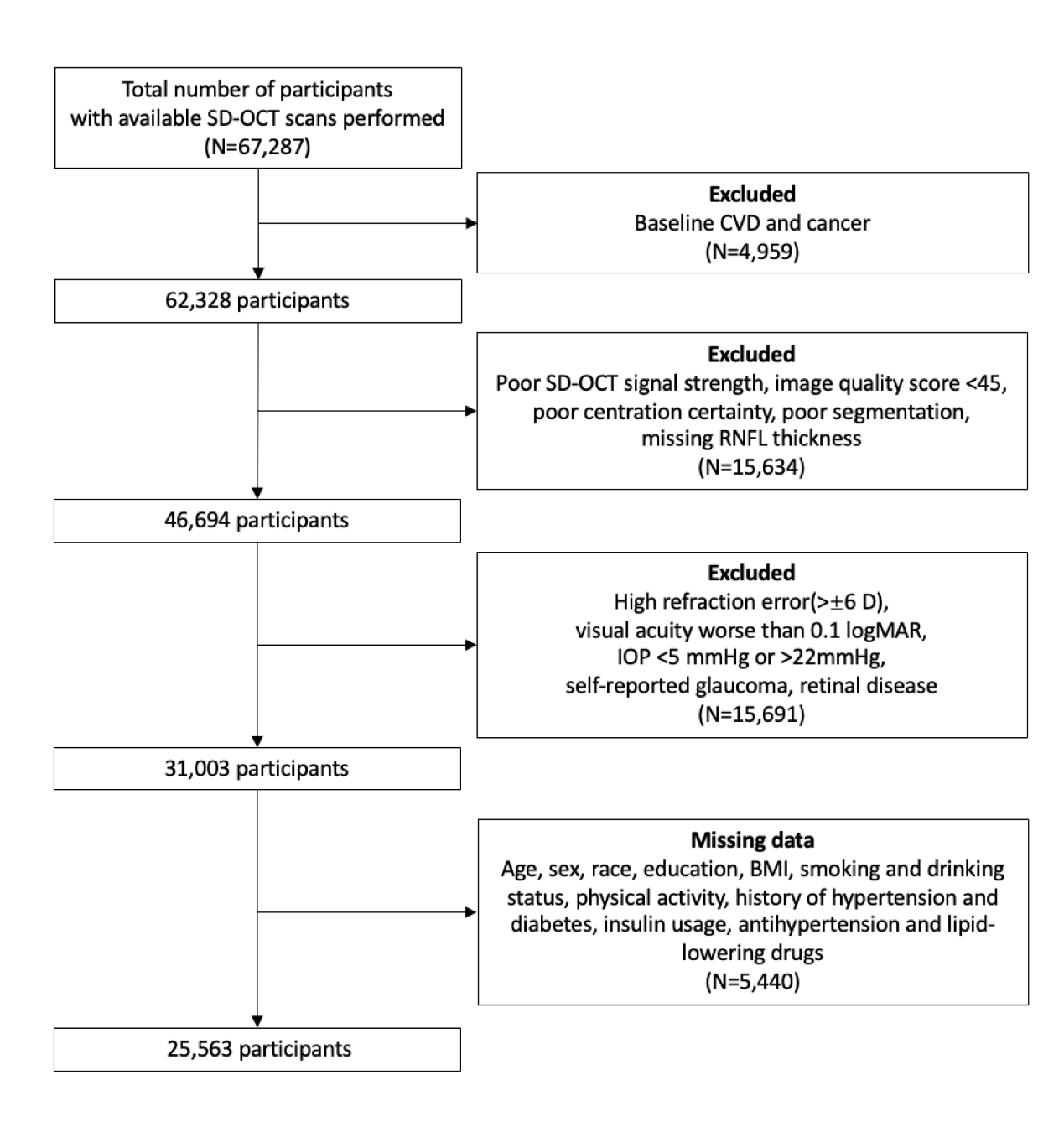
**

SD-OCT=spectral domain optical coherence tomography; CVD=cardiovascular disease; RNFL=retinal nerve fiber layer; IOP=intraocular pressure; BMI= body mass index.

**Figure S2.** Macular OCT retinal layers segmentation in one sample OCT image from the UK Biobank

**
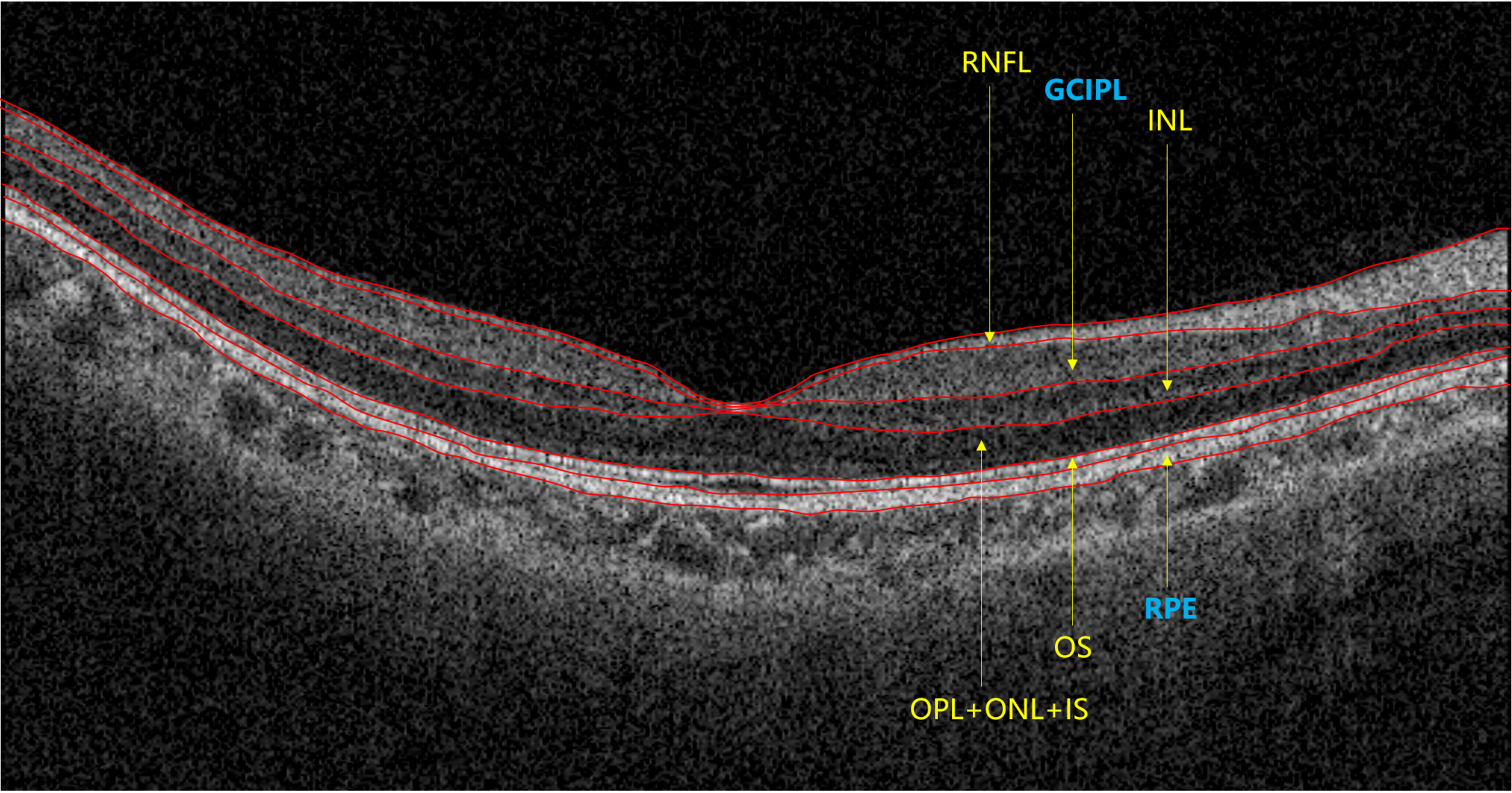
**

Macular RNFL refers to the innermost layer of the retina.

RNFL=retinal nerve fiber layer; GCIPL=ganglion cell-inner plexiform layer; INL=inner nuclear layer; OPL=outer plexiform layer; ONL=outer nuclear layer; IS=photoreceptor inner segment; OS=photoreceptor outer segment; RPE=retinal pigment epithelium.

**Table S1.** Definition of variables in touchscreen questionnaire, verbal interview, and inpatient records of diagnosis

| **Variable** | **Data category** | **Data field** | **Data code** |
| --- | --- | --- | --- |
| **Age at recruitment** | Population characteristics | 21022 |  |
| **Sex** | Population characteristics | 31 |  |
| **UK Biobank assessment center** | Recruitment | 54 |  |
| **Average total household income before tax** | Touchscreen questionnaire | 738 |  |
| **Townsend deprivation index** | Population characteristics | 189 |  |
| **Smoking status** | Touchscreen questionnaire | 20116 |  |
| **Alcohol drinker status** | Touchscreen questionnaire | 20117 |  |
| **Ethnic background** | Touchscreen questionnaire | 21000 |  |
| **Qualifications** | Touchscreen questionnaire | 6138 |  |
| **BMI (Body mass index)** | Physical measures | 21001 |  |
| **MET minutes per week for moderate activity** | Touchscreen questionnaire | 22038 |  |
| **MET minutes per week for vigorous activity** | Touchscreen questionnaire | 22039 |  |
| **logMAR, final (left)** | Eye measures | 5208 |  |
| **logMAR, final (right)** | Eye measures | 5201 |  |
| **Cylindrical power (left)** | Eye measures | 5086 |  |
| **Cylindrical power (right)** | Eye measures | 5087 |  |
| **Spherical power (left)** | Eye measures | 5085 |  |
| **Spherical power (right)** | Eye measures | 5084 |  |
| **Intra-ocular pressure,**  **corneal-compensated (left)** | Eye measures | 5262 |  |
| **Intra-ocular pressure,**  **corneal-compensated (right)** | Eye measures | 5254 |  |
| **Average retinal nerve**  **fibre layer thickness (left)** | Eye measures | 28500 |  |
| **Average retinal nerve**  **fibre layer thickness (right)** | Eye measures | 28501 |  |
| **Diabetes** | Hospital inpatients (ICD-10) | 41270 | E11-E14 |
|  | Touchscreen questionnaire | 6148 | 1 |
|  | Touchscreen questionnaire | 2443 | 1 |
|  | Verbal interview | 20002 | 1276, 1220, 1222, 1223, 1468, 1521, 1607 |
| **Cardiovascular diseases** | Hospital inpatients (ICD-10) | 41270 | I20-I25, I50, I60-I64 |
|  | Touchscreen questionnaire | 6150 | 1, 2, 3 |
|  | Verbal interview | 20002 | 1074, 1075, 1076, 1081,  1082, 1086, 1491, 1583 |
| **Hypertension** | Hospital inpatients (ICD-10) | 41270 | I10, I15 |
|  | Touchscreen questionnaire | 6150 | 4 |
|  | Verbal interview | 20002 | 1065, 1072 |
| **Glaucoma** | Hospital inpatients (ICD-10) | 41270 | H40, H42 |
|  | Touchscreen questionnaire | 6148 | 2 |
|  | Verbal interview | 20002 | 1277 |
| **Retinal diseases** | Hospital inpatients (ICD-10) | 41270 | H30-H36 |
|  | Touchscreen questionnaire | 6148 | 1, 5 |
|  | Verbal interview | 20002 | 1275, 1281, 1282, 1528 |

**Table S2.** Incidence rates of CVD Events in UK Biobank stratified by mRNFL thickness tertiles

| **Average mRNFL thickness** | **No. of CVD incidence** | **Person-Years Follow-up** | **Incidence Rate of CVD/1000**  **Person-Years** | **95% CI (%)** |
| --- | --- | --- | --- | --- |
| Highest Tertile  (29.94-53.43 μm) | 345 | 65,000 | 5.33 | 4.80-5.92 |
| Second Tertile  (26.51-29.93 μm) | 432 | 64,000 | 6.74 | 6.13-7.40 |
| Lowest Tertile  (12.53-26.50 μm) | 504 | 64,000 | 7.91 | 7.25-8.63 |
| P for trend |  |  |  | **<0.001** |

mRNFL=macular retinal nerve fibre layer; CVD=cardiovascular disease; CI=confidence interval.

**Table S3.** Baseline characteristics of participants in GDES stratified by incident CVD

| **Baseline Characteristics** | **Non-CVD group** | **CVD group** | ***P* value** |
| --- | --- | --- | --- |
| No. | 606 | 29 | - |
| Age (year) | 64.24 (7.64) | 71.71 (5.98) | **<0.001** |
| Female, No. (%) | 353 (58.15) | 9 (29.03) | **0.001** |
| Body mass index (kg/m^2^) | 24.43 (3.19) | 23.86 (2.03) | 0.326 |
| Systolic blood pressure (mmHg) | 133.31 (18.31) | 143.23 (18.34) | **0.003** |
| Diastolic blood pressure (mmHg) | 70.72 (9.70) | 71.13 (9.75) | 0.820 |
| Duration of diabetes (year) | 9.26 (6.83) | 13.62 (8.16) | **<0.001** |
| HbA1c (%) | 6.83 (1.28) | 8.18 (1.56) | **<0.001** |
| Total cholesterol (mg/dl) | 183.77 (38.50) | 179.28 (50.29) | 0.534 |
| High-density lipoprotein cholesterol (mg/dl) | 50.84 (15.08) | 49.07 (16.13) | 0.527 |
| Low-density lipoprotein cholesterol (mg/dl) | 116.94 (35.39) | 106.19 (30.42) | 0.097 |
| Smoking status, No. (%) |  |  | 0.269 |
| Never | 479 (83.60) | 18 (75.00) |  |
| Ever/Current | 94 (16.40) | 6 (25.00) |  |
| Drinking status, No. (%) |  |  | **0.001** |
| Never | 513 (89.53) | 16 (66.67) |  |
| Ever/Current | 60 (10.47) | 8 (33.33) |  |
| Insulin usage, No. (%) |  |  | **<0.001** |
| No | 487 (80.23) | 16 (53.33) |  |
| Yes | 120 (19.77) | 14 (46.67) |  |
| Intraocular pressure (mmHg) | 16.38 (2.82) | 16.26 (2.70) | 0.812 |
| Axial length (mm) | 23.66 (1.08) | 24.43 (1.31) | **<0.001** |
| Image quality score | 61.59 (9.11) | 60.67 (7.50) | 0.672 |

Data are presented as No. (percentage) or mean (standard deviation [SD]).

CVD=cardiovascular disease; GDES=Guangzhou Diabetes Eye Study.

**Table S4.** Distribution of baseline peripapillary RNFL (pRNFL) of participants in GDES stratified by incident CVD

| **pRNFL** | **Non-CVD group** | **CVD group** | ***P* value** |
| --- | --- | --- | --- |
| Average pRNFL (μm) | 110.41 (12.07) | 91.21 (20.14) | **<0.001** |
| Superior pRNFL (μm) | 130.77 (21.17) | 104.12 (27.40) | **<0.001** |
| Inferior pRNFL (μm) | 143.35 (23.28) | 111.46 (38.90) | **<0.001** |
| Nasal pRNFL (μm) | 87.07 (19.52) | 76.29 (20.67) | **0.004** |
| Temporal pRNFL (μm) | 80.28 (16.67) | 73.06 (18.31) | **0.024** |

Data are presented as mean (standard deviation [SD]).

pRNFL=peripapillary retinal nerve fibre layer; CVD=fatal and non-fatal cardiovascular disease; GDES=Guangzhou Diabetes Eye Study.

**Table S5.** Summary of previous cross-sectional studies on RNFL and cardiovascular diseases

| **Study** | **Exposure** | **Outcome** | **Primary findings** |
| --- | --- | --- | --- |
| Kim et al. [42] | Localized pRNFLT | Cerebral small vessel disease (SVD) | Thinning in cerebral SVD |
| Wang et al. [9] | Localized pRNFLT | Stroke | Thinning in stroke |
| Kanar et al. [10] | pRNFLT | Heart failure | Thinning in serious heart failure |
| Neoh et al. [12] | pRNFLT | Coronary artery disease (CAD) | Thinning in CAD |
| de Aguiar et al. [41] | pRNFLT, mRNFLT | Congenital heart disease (CHD) | Thinning in CHD |
| Xu et al. [44] | Localized pRNFLT | Hypertension | Thinning in hypertension |
| Wang et al. [43] | pRNFLT | Carotid artery stenosis | Thinning in carotid artery stenosis |
| Shin et al. [13] | Localized pRNFLT | ASCVD risk score | Thinning in higher ASCVD risk score |
| Zhang et al. [15] | pRNFLT | Ideal cardiovascular health | No correlation |
| Lamparter et al. [14] | pRNFLT | Cardiovascular factors | No correlation |

pRNFLT=peripapillary retinal nerve fiber layer thickness; mRNFLT=macular retinal nerve fiber layer thickness; ASCVD=atherosclerotic cardiovascular disease.
